# Generation, annotation and analysis of ESTs from *Trichoderma harzianum *CECT 2413

**DOI:** 10.1186/1471-2164-7-193

**Published:** 2006-07-27

**Authors:** Juan Antonio Vizcaíno, Francisco Javier González, M Belén Suárez, José Redondo, Julian Heinrich, Jesús Delgado-Jarana, Rosa Hermosa, Santiago Gutiérrez, Enrique Monte, Antonio Llobell, Manuel Rey

**Affiliations:** 1 IBVF-CIC Isla de la Cartuja, CSIC/Universidad de Sevilla. Avda. Américo Vespucio s/n. 41092, Sevilla, Spain; 2 Newbiotechnic, S. A. (NBT). Parque Industrial de Bollullos A-49 (PIBO). 41110, Bollullos de la Mitación. Sevilla, Spain; 3 Spanish-Portuguese Center of Agricultural Research (CIALE), Departamento de Microbiología y Genética, Universidad de Salamanca, Edificio Departamental, lab 208, Plaza Doctores de la Reina s/n, 37007, Salamanca, Spain; 4 Area of Microbiology. Escuela Superior y Técnica de Ingeniería Agraria. Universidad de León, Campus de Ponferrada. Avda. Astorga s/n. 24400, Ponferrada, Spain

## Abstract

**Background:**

The filamentous fungus *Trichoderma harzianum *is used as biological control agent of several plant-pathogenic fungi. In order to study the genome of this fungus, a functional genomics project called "TrichoEST" was developed to give insights into genes involved in biological control activities using an approach based on the generation of expressed sequence tags (ESTs).

**Results:**

Eight different cDNA libraries from *T. harzianum *strain CECT 2413 were constructed. Different growth conditions involving mainly different nutrient conditions and/or stresses were used. We here present the analysis of the 8,710 ESTs generated. A total of 3,478 unique sequences were identified of which 81.4% had sequence similarity with GenBank entries, using the BLASTX algorithm. Using the Gene Ontology hierarchy, we performed the annotation of 51.1% of the unique sequences and compared its distribution among the gene libraries. Additionally, the InterProScan algorithm was used in order to further characterize the sequences. The identification of the putatively secreted proteins was also carried out. Later, based on the EST abundance, we examined the highly expressed genes and a hydrophobin was identified as the gene expressed at the highest level. We compared our collection of ESTs with the previous collections obtained from *Trichoderma *species and we also compared our sequence set with different complete eukaryotic genomes from several animals, plants and fungi. Accordingly, the presence of similar sequences in different kingdoms was also studied.

**Conclusion:**

This EST collection and its annotation provide a significant resource for basic and applied research on *T. harzianum*, a fungus with a high biotechnological interest.

## Background

*Trichoderma *is a fungal genus that includes cosmopolitan fungi able to colonize different substrates under diverse environmental conditions. One of the most significant ecological niches occupied by *Trichoderma *species is the plant rhizosphere, which is effectively colonized due to the capacity of these fungi to interact with plants and compete with other soil organisms [1]. This ability is the result of a long period evolution in which biological mechanisms for attacking other microorganisms and for enhancing plant growth have developed in *Trichoderma *[2]. The biocontrol activity of *Trichoderma *depends on its metabolic versatility and secretory potential, which are responsible for the production of large amounts of highly diverse hydrolytic enzymes involved in the degradation of fungal cell walls [3]. Since *Trichoderma *species are efficient antagonists of other fungi and due to their ubiquity and rapid substrate colonization, they have been commonly used as biocontrol organisms for agriculture, and their enzyme systems are widely used in industry [4].

The *Trichoderma *genome, although it is a fungal genus of high biotechnological value, has been poorly surveyed compared to other microorganisms. A structural genomics project, carried out by the U.S. Joint Genome Institute [5] has provided the first version of the full genome sequence of the *T. reesei *strain QM 9414, an isolate without known biocontrol abilities, but with industrial interest. Additionally, the functional genomics EU-funded project "TrichoEST" [6] was undertaken by an International Consortium comprised of academic institutions and enterprises. The aims were to identify genes and gene products from twelve strains with biotechnological value from different *Trichoderma *species [7]. In this project, the antagonistic strain *T. harzianum *CECT 2413 was selected representing the *T. harzianum *biotype.

In this work, mRNA populations from *Trichoderma *transcribed among others, under mycoparasitic and nutrient stress conditions, trying to simulate some of the environmental conditions that take place in the soil, were cloned as cDNAs and were the origin of expressed sequence tags (ESTs). This strategy has been used as an efficient and economical approach for large-scale gene discovery, to explore gene regulation patterns and to identify differentially regulated genes. During the last years, a large number of ESTs have been generated from several filamentous fungi and Oomycetes, including among others *Gibberella zeae *[8], *Magnaporthe grisea *[9], *Mycosphaerella graminicola *[10], *Phytophthora parasitica *[11], *Uromyces fabae *[12] or *Ustilago maydis *[13,14].

There are several previous studies involving EST approaches that have been carried out in *Trichoderma *species. In the first one [15], the metabolism of *T. reesei *QM 9414 was studied focusing on the anaerobic and aerobic degradation of glucose. Their genomic work produced the sequence of 2,835 randomly selected cDNAs corresponding to 1,151 unique transcripts and the complete mitochondrial genome of *T. reesei*. Their complete EST database and the details on the experimental procedure and results are available on line [16]. In another study [17], 18,000 ESTs from a sole cDNA library were sequenced and 5,131 unique sequences were obtained from *T. reesei *QM6a. They also detected twelve enzymes involved in biomass degradation, performed microarray expression analysis and found that these enzymes were transcriptionally regulated [17].

Diener *et al *[18] identified genes from *T. reesei *QM6a involved in protein processing and secretion. A total of 21,888 ESTs from two different cDNA libraries were sequenced, corresponding to 7,943 unique transcripts. Liu and Yang [19], working with a *T. harzianum *unknown strain, obtained 3,298 ESTs (1,740 unique transcripts) from a single cDNA library. In the most recent study, 2,047 ESTs (457 unique sequences) were retrieved using a subtractive strategy. Two cDNA subtraction libraries were used in order to identify genes that were differentially expressed in response to secretion stress in *T. reesei *Rut-C30 [20].

In the present study we report the analysis of 8,710 ESTs obtained from *T. harzianum *CECT 2413. These sequences were derived from eight different cDNA libraries that had been made combining different growth conditions. Overall, 3,478 unique transcripts were identified and GO-terms were assigned. In addition, the relative abundance of ESTs provided a measure of gene expression. We identified the putative secreted proteins and also performed a comparative analysis among our dataset and the other collections of ESTs from *Trichoderma *species. Finally, the *T. harzianum *ESTs were compared to genomic sequence databases from several animals, plants and fungi.

## Results

### EST sequence determination and analysis

Different conditions were used to build specific and mixed cDNA libraries for the "TrichoEST" project as seen in the Methods section. Eight different libraries were made (Table 1) and EST sequences were produced (Table 2). A total of 7,283 sequences were identified as having high quality by Phred [21] (quality values greater than 20) from the 8,710 sequencing reactions (83.6%). The average sequence length was 566 nucleotides, and approximately 79.8% of the ESTs were longer than 400 nucleotides, while 7.2% of the sequences were shorter than 100 nucleotides.

ESTs were generated from different libraries with the expectation that more unique genes would be identified from moderate levels of sequencing from diverse libraries rather than deep sequencing of a single library. The combined ESTs for all libraries clustered into 889 multisequence contigs, with 2,589 singlets yielding an estimate of 3,478 unique sequences. These unique sequences are listed in the additional file 1. The ESTs that are contained in each contig are listed in the additional file 2.

The distribution of ESTs across multiple libraries was assessed as a measure of uniqueness of gene concurrence across libraries. No contig contained sequences that were from seven or eight libraries, and only nine unique sequences were present in six libraries. Most of the contigs contained sequences found in one to three libraries, with the largest category being contigs represented by clones from two libraries (393 clones). This pattern is mainly due to the fact that many of the contigs contain only a small number of ESTs. Considering only those contigs representing 20 or more ESTs, it is found that most of the genes are usually represented in multiple libraries.

### Functional annotation and analysis

Unique sequences were assigned functions according to gene ontology (GO) terms [22] based on BLAST definitions using the program Blast2GO [23]. GO categories were assigned to 1,776 of the 3,478 predicted unique sequences (51.1%). Later, we used a locally implemented AmiGO browser in order to examine the representation of genes across different functional categories. Our AmiGO browser is publicly available [24]. However, in this paper we only cite those libraries that provided more than 300 unique sequences: L02, L03, L06 and L10 (Table 3).

The gene distribution in the main ontology categories was studied and the percentages of unique sequences with assigned GO terms that fell into these categories were calculated. For this purpose, we considered 100% as the total number of unique sequences from each of the libraries that possessed an assigned GO term in each of the three organizing principles of GO (Biological Process, Molecular Function and Cellular Component) [22]. It must be taken into account that these percentages do not add up to 100% because many deduced proteins can have more than one GO assigned function. Gene distribution was very similar across the libraries that came from *Trichoderma *grown in liquid media (L02, L03 and L10). However, differences were found in L06, made from *Trichoderma *grown in solid media (solid minimal medium containing 0.1% glucose or PPG medium). For example, in L06 the percentage of GO terms in the following categories was significantly higher than in the other libraries: "metabolism" (92.9%), "biosynthesis" (56.4%), "cellular metabolism" (85.8%), "macromolecule metabolism" (64.4%), "primary metabolism" (80.6%), "nucleic acid binding" (22.8%), "structural molecule activity" (27.6%) and "intracellular" (93.0%). Additionally, in L06 a lower percentage was found in other categories like "catalytic activity" (46.5%), "hydrolase activity" (14.5%), "extracellular region" (0.6%) or "membrane" (15.4%).

### Identification of putative secreted proteins

*T. harzianum *is the source of a number of secreted proteins produced for various industrial applications. To identify potential secreted proteins we used SignalP 3.0 [25] in order to search for predicted proteins with a signal peptide. We found that 800 of these predicted proteins (23.0%) possessed this putative signal peptide (see additional file 1).

### Exploration of more abundantly expressed genes

Sequencing of random cDNA clones allows studies of mRNA abundance. Thus, analysis of the frequency of specific ESTs that form individual contigs can provide information with respect to the expression levels of particular genes under different experimental conditions [9,10]. Table 4 displays the total number of contigs made up of 20 or more ESTs together with the originating libraries. The data illustrate the functional diversity of these highly expressed unique sequences with apparently no particular functional category dominating the analysis. However, at this point it must be considered that a significant amount of hypothetical and/or unassigned-function proteins were also detected. As expected, a number of housekeeping genes involved in protein translation, carbon metabolism and energy production were identified, as some of the hits corresponded to genes like the translation elongation factor 1α, histones H3 and H4, polyubiquitin, glyceraldehyde 3-phosphate dehydrogenase or ribosomal proteins. The most abundantly represented gene in the total collection (T34C463, 95 ESTs) was similar to a hydrophobin from *Hypocrea jecorina *(anamorph *T. reesei*). Hydrophobins are small molecular weight proteins of fungal origin that can play roles in diverse physiological processes including adhesion, development and pathogenesis [26,27].

We also found genes probably involved in the synthesis of thiamine (vitamin B1): T34C494 (46 ESTs) and T34C829 (24) were similar to the NMT1 protein and T34C811 (28) was similar to the THI4 protein, a thiazole biosynthetic enzyme. Additionally, we also detected sequences similar to an ADP/ATP carrier protein (T34C470), the ATP synthase protein 9 (T34C216) [28], a peptide involved in stress response (T34C759) and a cyclophilin (T34C418).

### Comparison to the nr database and InterProScan annotation

Sequence comparison using the BLASTX algorithm against the NCBI non redundant (nr) database allowed the identification of 2,832 unique sequences (81.4%). Thus, 646 sequences did not exhibit significant similarity (E-value < 10^-5^) to genes in the nr database.

Additional information on the ESTs was obtained by protein-signature scanning. InterProScan was used for sequence comparison to the InterPro database [29]. This database contains signature information from Hidden Markov models, regular expressions, fingerprints and profiles for protein families, and domains from public domain database projects including Pfam [30], PROSITE [31], ProDom [32], PRINTS [33], TIGRFAMS [34] and SMART [35]. The submission facilitated the annotation of 3,331 (95.8%) unique sequences with significant protein signatures. Within them, 2,006 (57.7%) sequences contained associated InterPro (IPR) numbers. Of these 2,006 sequences, 76 had not been annotated during the sequence comparison against the NCBI nr database. These data are included in the additional file 1.

### Comparison with other *Trichoderma *collections of ESTs

We used the tBLASTX algorithm [36] in order to study the presence of similar sequences in the collections of ESTs from *Trichoderma *publicly available. At the E-value < 10^-10 ^level, the highest percentages of similar sequences were found in three collections from *T. reesei*: 44.7% [18], 40.5% [17] and 25.1% [15]. Unexpectedly, only 21.6% of similar sequences was found in the related to biocontrol collection obtained from *T. harzianum *[19]. Finally, a very low percentage (4.5%) of similar sequences was found with the ESTs recently obtained from *T. reesei *following a subtractive strategy [20].

### BLAST analysis to species sequence datasets

We used the BLASTX algorithm [36] to identify ESTs with sequence similarity to the protein sequences derived from the genomic sequence datasets of 15 eukaryotic species (two animals, two plants and eleven fungi). We used an E-value < 10^-5 ^as indicative of sequence similarity significance. In these comparisons it is important to note that the *T. harzianum *sequences do not represent the complete genome.

All unique sequences from *T. harzianum *were queried against the sequence datasets. A table listing the number of unique sequences possessing a top hit below the E-values of 10^-5^, 10^-25^, 10^-50^, 10^-75^, and 10^-100 ^was produced (Table 5). Of the 3,478 unique sequences from *T. harzianum*, the highest proportion of similar sequences was found in filamentous fungi. At the 10^-5 ^level, the percentages were: *T. reesei *(75.2%), *Fusarium graminearum *(71.7%), *Neurospora crassa *(66.4%), *M. grisea *(63.4%) and *Aspergillus nidulans *(59.1%). These proportions decreased in the basidiomicete *U. maydis *(42.0%) and *Cryptococcus neoformans *(35.9%) and also in the yeasts *Candida albicans *(41.4%), *Schizosaccharomyces pombe *(39.2%) and *Saccharomyces cerevisiae *(39.0%). At this level, a total percentage of an 80.2% of the unique sequences had sequence similarity with at least one of the fungal datasets. The number of ESTs plus contigs with similarity to sequences of plants was slightly higher (33.3% in total, 32.5% for *Arabidopsis thaliana*, 29.3% for *Oryza sativa*) than what was found in animals (31.3% in total, 25.8% for *Drosophila melanogaster*, 25.1% for *Caenorhabditis elegans*). A total of 2,793 unique sequences (80.3%) showed sequence similarity with at least one species with an E-value < 10^-5^. Logically, these percentages decreased as the E-value increased (Table 5). For instance, at the E < 10^-100 ^level, 11.6% of the sequences had similar sequences in *T. reesei*, 5.0% in *F. graminearum *and 4.0% in *A. nidulans*, being the rest of the genomes not significantly represented.

**Figure 1:**
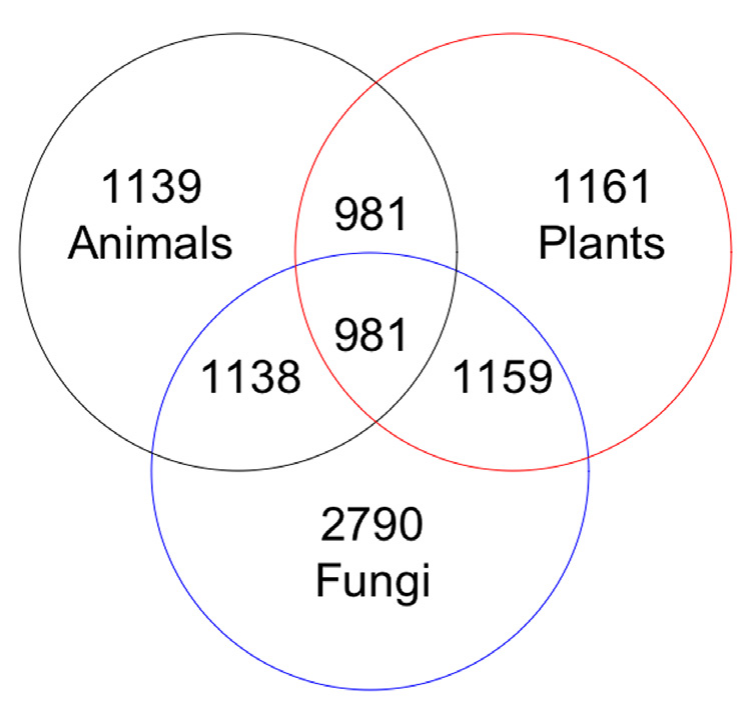
Venn diagram of the *Trichoderma *unique sequences (3,478) with similar sequences in other fungi, plants and animals. The numbers for the different taxa are shown at the level E-value < 10^-5^.

Further analysis of the *T. harzianum *unique sequences grouped these sequences based upon the eukaryotic taxa in which sequence similarity was found. The classification of unique sequences conserved across all eukaryotes was defined by at least one similar sequence in each of the fungi, plants and animals. Under these criteria, 981 of the 2,793 unique sequences (35.1%) that showed sequence similarity (*E*-value < 10^-5^) with at least one organism, were present in all eukaryotic taxa (Figure 1). These sequences are listed in the additional file 3.

Moreover, 1,138 unique sequences were found in genomes from fungi and animals (not present in plants) and a slightly higher number of genes (1,159) was shared between fungi and plants (not present in animal genomes). Furthermore, the number of unique sequences with similarity to the fungal and plant genomes but not the animal genomes was 178. Meanwhile, the number of unique sequences with similar sequences in the fungal plus in either of the two animal, but not in the plant genomes was 157 (Figure 1). The *T. harzianum *unique sequences found to have similar sequences only in fungal species totalled 1,474 (42.4%). These sequences are also listed in the additional file 3.

The number of orphan unique sequences (those showing no similarity with any other organism or the nr database) was 487 (14.0%). However, the number of unique sequences lacking a similarity to sequences in the nr database was 646 (18.8%). Thus, after our cross-species identification of similar sequences, we reduced the number of orphan genes by another 159 unique sequences.

## Discussion

We are investigating the genome of *T. harzianum *CECT 2413, a strain with a great interest in biotechnology and biological control [37,38]. EST sequencing provides information about functional identification of genes, gene structure and gene expression patterns. Like in other recent studies carried out in fungi [9,39], a strategy of sequencing a variety of different libraries was used to maximize the number of unique genes. The approach based on the construction of cDNA libraries from a mixture of conditions was successfully used in *T. reesei *by Foreman *et al*. [17]. The *Trichoderma *growth conditions for the different cDNA libraries were mainly chosen in order to simulate *in vitro *some aspects of the biocontrol process occurring in the soil environment like mycoparasitism [7]. This strategy has proved to be successful in order to identify genes involved in the biocontrol such as *ThPTR2 *(EST L03T34P074R06935), a peptide transporter recently studied by our group that was highly expressed when *Trichoderma *was interacting with the plant-pathogenic fungus *Botrytis cinerea *[40]. Additionally, similar findings have been obtained for other genes like *erg1*, involved in the biosynthesis of terpenes [41] or the proteases *pra1 *[42] and *p6281 *[43].

Overall, we searched in our collection of ESTs for unique sequences encoding cell wall degrading enzymes, which could be involved in the mycoparasitism. For this purpose we looked at the BLAST definitions and found several unique sequences with putative chitinase (6 unique sequences), glucanase (30) or protease (54) activities. This isoenzyme multiplicity has been described before by different groups and reported by our team in a study where several isoenzymes with glucanase, protease or chitinase activities were detected in different *Trichoderma *species, including *T. harzianum *CECT 2413 [44].

Sequence redundancy within our study was 56.4%. This level of redundancy was lower than in the two comparable studies carried out in *T. reesei*: 71.5% [17] and 59.4% [15]. In the first one, 18,000 ESTs were sequenced from a single cDNA library made from a mixture of more than 20 different growth conditions. However, in the second one [15], a unique growth condition was used and the number of sequenced ESTs was also lower (2,835). The subtractive study [20] had a sequence redundancy of 77.7%. Finally, the remaining study is not comparable since a separate assembly process was used for each library [18]. Taking into account that sequence redundancy increases with the total number of sequenced ESTs, we consider that our strategy, designed in order to maximize the number of unique genes without increasing in excess the number of sequences, worked properly.

Overall, 3,478 unique sequences were identified in *T. harzianum *CECT 2413, which represents a significant portion of the genome. According to recent available data [45], the number of genes in the *T. reesei *genome (34.5 Mb) is very close to 10,000. This is similar to what has been found in other filamentous fungi. Thus, our collection of genes would represent about one third of the total.

BLASTX searches indicated that 81.2% of the unique sequences had sequence similarity to an entry in the NCBI nr database. This percentage is quite similar to what has been recently found in a similar study in *Aspergillus niger *(83.0%) [46], but it is much higher than what was found in other recent EST studies carried out in other fungi like *Conidiobolus coronatus *(58%) [47], *Phakospora pachyrhizi *(48%) [48] and *U. maydis *(57–59%) [13,14]. New sequence data from complete genome projects from several fungi had made possible this increase although a high proportion of the hits remains annotated as hypothetical proteins.

The degree of annotation was extended through the identification of protein motifs, using InterProScan searches of the InterPro database. This extension resulted in annotation of a 95.8% (3,331) of the unique sequences, including 2,006 with InterPro annotation. This percentage is again much higher than the one found in *U. maydis *(69.4%) [13].

As seen before, in a number of cases clones that did not cluster (considered as unique sequences) displayed sequence similarity to the same protein. Several factors can account for this. Among them, (i) the clones are different genes that are homologs of the same protein, (ii) the clones align with different regions of the same search hit but do not overlap (or have too small of an overlap) with each other, and (iii) the clones represent different splice variants of the same gene. We searched for alternative transcript forms by looking carefully inside each contig but apparently, none of them were detected in the present study. They have been recently found in other filamentous fungi like *M. grisea *[9] and *Fusarium verticillioides *[39], although perhaps the most extensive genome-wide survey on alternative splicing in a fungus comes from the basidiomycete *C. neoformans *serotype D [49], where this mechanism was found in 4.2% of the genes. Additionally, there are at least two studies where alternative splicing has been described in *Trichoderma *species. In the first one, carried out also in *T. harzianum *CECT 2413, two alternative forms of a glucoamylase gene were detected in different growth conditions [50]. In the second one, the authors found two different mRNA species from two chitinases (chi18-3 and chi18-13), depending also on the culture media where *T. atroviride *had been previously grown [51].

### EST abundance

The analysis of the relative abundance of individual ESTs that make up unique sequences (contigs) from different libraries can be used as a first indicator of transcript abundance. We identified a number of unique sequences generated from 20 or more ESTs (Table 4). Apparently, no particular gene family was predominant. This agrees with some previous works [10,18], but it is in contrast with other analysis where particular classes of genes (e.g., encoding ribosomal proteins) dominated these frequency tables [8,14]. However, numerous housekeeping genes were detected as expected.

A unique sequence similar to the hydrophobin II from *H. jecorina *was the most represented gene. A similar sequence (contig1201) was also found as one of the most expressed genes in the library LT002 from *T. reesei *[18]. However, we have not found another possible hydrophobin in a similar frequency table made in any other fungus including *T. harzianum *[19]. In a recent study [52], it was found that the *T. reesei *hydrophobins I and II had a role in hyphal development and sporulation, respectively. Among the most highly expressed genes we also detected sequences that could be involved in thiamine biosynthesis, similar to the NMT1 and THI4 proteins respectively. THI4 is a thiazole biosynthetic enzyme that had been previously found (contig437) as highly expressed in *T. harzianum *[19]. However, both genes could also be involved in other processes [53]. In *S. cerevisiae *THI4 appears to be a dual function protein involved in thiazole biosynthesis and tolerance to mitochondrial DNA damage [54].

A putative cyclophilin was also identified as one of the most abundant transcripts. Cyclophilins include the binding proteins of the cyclic peptide cyclosporin A, they possess peptidyl-prolyl cis-trans isomerase activity *in vitro *and can play roles in protein folding and transport, RNA splicing and the regulation of multi-protein complexes in cells [55]. One similar sequence was also found as one of the most expressed genes (contig429) in the EST collection from *T. harzianum *[19]. So far, cyclophilins have been more studied in yeasts [56,57] than in filamentous fungi [58,59].

### GO terms

The percentage of assigned GO-terms (51.1%) was slightly higher than in one similar study in *A. niger *[46]. As far as we know, this is the first time that the program Blast2GO [23] has been used for this purpose. Clear differences were found in the distribution of the GO terms among the gene library L06 and the other three (L02, L03 and L10). This could be explained considering that L06 was the only library among them made using mRNA from *Trichoderma *growing in solid medium. However, it must be also considered that these differences could arise more from their different composition than from the solid or liquid nature of the media. The liquid media included different stress-related growth conditions like nitrogen or carbon starvation, chitin or fungal cell walls as sole carbon source whereas the solid medium covered only carbon starvation.

We identified 800 predicted proteins with a putative signal peptide. Gene Ontology annotation categorized only 80 of the predicted proteins as "extracellular", included in the GO terms "extracellular matrix" (GO:0031012) and "extracellular region" (GO:0005576) (see [24]). However, it must be taken into account that we were able to assign a GO term to 51.1% of the unique sequences. The percentage of predicted proteins with signal peptide (23.0%) was almost identical to what was found in *A. niger *(23.4%) [46] but lower than in *T. reesei *(33%), using the same algorithm [18]. These differences could be related to the different growth conditions from which the cDNA libraries were made or to the fact that they are different species.

### Comparison with other *Trichoderma *collections of ESTs

We found that only 21.6% of the unique sequences had similar sequences in the related to biocontrol collection obtained from *T. harzianum *[19]. A higher number of similar sequences was found in the other comparable collections from *T. reesei*, specifically in the collection of ESTs involved in protein processing and secretion [18]. These results were unexpected because in principle, a highest number of similar sequences should have been found in the EST collection related to biocontrol [19] than in the *T. reesei *collections. The lack of information on the strain and the growth conditions in which that EST collection for *T. harzianum *was obtained [19] makes difficult to explain this fact. As for the differences in the presence of similar sequences between the three comparable collections from *T. reesei*, it is logical that the lowest percentage of similar sequences was found in a collection obtained in very different growth conditions, with glycerol as sole carbon source [15], than the ones used in our study.

### BLAST analysis to species sequence datasets

The unique sequences were compared to the genomes of eleven different fungi, two animals (*C. elegans *and *D. melanogaster*) and two plants (*A. thaliana *and *O. sativa*). Within the total of 2,793 unique sequences (80.3%) that showed sequence similarity with at least one species (at E-value < 10^-5^), 2,616 (93.7%) had a similar sequence in the *T. reesei *complete genome. It must be considered that many gaps are still present in the current publicly available version (v1.0) of this genome [5]. The new version (v2.0) containing very few gaps will be available soon [45] and will allow us to further study the unique sequences that could be found in *T. harzianum *but not present in *T. reesei*.

Behind *T. reesei*, the ascomycete *F. graminearum *possessed the largest number of similar sequences to the *T. harzianum *unique sequences. This is not surprising due to the close taxonomic relationship between the *Trichoderma *and *Fusarium *genera, because their teleomorphs are located in the close families Nectriaceae and Hypocreaceae, respectively [60].

In the species-by-species comparison, 981 (28.2%) unique sequences were present in all the eukaryotic taxa. A similar percentage (29%) was found in a similar study in *U. maydis *[13]. These 981 clones constitute a 35.1% of the unique sequences (2,793) that showed sequence similarity (at the level E-value < 10^-5^) with at least one organism (animals, plants or fungi). This number is perhaps slightly low because it has been described that about 40% of the total genes in an eukaryotic genome may be shared with other eukaryotes of different kingdoms, although this does not mean that they are all essential genes [61,62]. This slightly low percentage could be due to the fact that our EST data set constitutes only a portion of the genome.

*T. harzianum *sequences found to have similar sequences only in fungal species totalled 1,474 (42.4%). This percentage is much higher than what was found in *U. maydis *(12.3%) [13]. There may be genes among these that have retained phylogenetic signatures dating to the separation of fungi and animals, or genes with signatures representing further changes leading to the current state of *T. harzianum*.

## Conclusion

The 8,710 ESTs identified in this study represent the major attempt so far to define the *T. harzianum *gene set and represent about 3,478 genes. Thus, these data dramatically increase the number of identified *T. harzianum *genes. The clone collection offers a base for expression profiling, enabling the identification of genes involved in specific physiological processes. The application of these results is not only of a great interest in biocontrol of plant-pathogens but also in the searching of genes with high biotechnological value.

## Methods

### Fungal strains

*T. harzianum *CECT 2413 (Spanish Type Culture Collection, Valencia, Spain) was used in this study. *Phytophthora syringae *CECT 2351, *B. cinerea *CECT 2100, *Penicillium aurantiogriseum *IMI 374515 (International Mycological Institute, Egham, UK) and *Rhizoctonia solani *CECT 2815 were used as a source to obtain fungal cell walls. The fungal strains were maintained on potato dextrose agar (PDA, Difco Becton Dickinson, Sparks, MD).

### cDNA libraries construction

A set of more than eight different conditions, most of them designed in order to simulate biocontrol processes were used to build specific and mixed cDNA libraries. The following libraries were made for the "TrichoEST" project: L02, L03, L05, L06, L08, L10, L11 and L15 (Table 1).

For the L02 library the biomass was obtained following a two-step liquid culture procedure. First, *T. harzianum *CECT 2413 was grown in a minimal medium [63] (MM: 15 g/l NaH_2_PO_4_, 5 g/l (NH_4_)_2_SO_4_, 600 mg/l CaCl^.^2H_2_O, 600 mg/l MgSO_4_^.^7H_2_O, 5 mg/l FeSO_4_, 2 mg/l CoCl_2_, 1.6 mg/l MnSO_4_, 1.4 mg/l ZnSO_4_), containing 2% glucose as carbon source, in baffled flasks at 25°C and 160 rpm for two days. Biomass was harvested, rinsed twice with sterile distilled water and transferred to MM [63] containing 0.5% of a 1:1 mixture of fungal cell walls from *P. syringae *and *B. cinerea*. It was incubated during 9 h.

For the L03 library, *T. harzianum *was grown in potato dextrose broth (Difco Becton Dickinson), in baffled flasks at 25°C and 160 rpm for two days. Biomass was treated as indicated above and transferred to MM under the following conditions in separate cultures: (i) 0.1% glucose, (ii) 1.5% chitin (Sigma, St. Louis, MO), (iii) a 100-fold increase in the concentration of metals or (iv) 1% of a 1:1 mixture of fungal cell walls from *P. aurantiogriseum *and *B. cinerea*. The cultures were incubated under these conditions for 8 and 12 h.

No pre-culture was performed when L05 library was made. *T. harzianum *was directly grown in MM containing 5% glucose during 30 h.

For the L10 library, *T. harzianum *was grown in MM containing 4% glucose, in baffled flasks at 25°C and 160 rpm for 36 h. Biomass was treated as indicated above and transferred to MM under the following conditions in separate cultures: (i) (ii) MM containing 1.5% chitin during 8 and 20 h, respectively (iii) MM buffered at pH 2.5 with HCl containing 1.5% chitin (Sigma, St. Louis, MO) during 8 h, or (iv) MM containing 2% glucose, in nitrogen starvation conditions (50 mg/l ammonium sulphate), during 8 h.

In order to make the L15 library no pre-culture was performed. *Trichoderma *was directly grown in MM containing 0.1% polygalacturonic acid, 0.1% carboxymethylcellulose, 0.1% pectin, 0.1% xylan and 0.2% glucose, at 28°C during 36 h.

Growth conditions for the remaining libraries involved solid media. For the L06 library, the biomass was obtained after *Trichoderma *was cultured (1.5 × 10^6 ^spores onto cellophane sheets) in solid MM containing 0.1% glucose or PPG medium (2% dextrose, 2% smashed potatoes, 2% agar) plates. For the L08 library, the biomass was obtained after *Trichoderma *was cultured during three days (1.5 × 10^6 ^spores onto cellophane sheets) in solid MM containing 0.02% colloidal chitin as carbon source.

Finally, for the L11 library, plates of MM containing 0.2% glucose were inoculated with one 0.5-cm mycelial plugs of *R. solani*. After 3.5 days of incubation, *R. solani *was covered with a cellophane sheet and then, one plug of *T. harzianum *was inoculated on the top of the cellophane, in the other side of the plate, 5 cm far each other. After 3.5 days of growth at 25°C, mycelia were recovered from the *Trichoderma *overgrowth zone

In all cases, mycelia were harvested, rinsed twice with sterile distilled water, freeze-dried and kept at -80°C until RNA extraction. RNA was extracted from the mycelia by grinding them with a mortar and pestle under liquid nitrogen and extracted using TRIZOL^® ^reagent (Invitrogen Life Technologies, Carlsbad, CA) according to the manufacturer's instructions. After total RNA extraction, an equal amount of RNA from the different growth conditions was mixed and mRNA was purified using poliA columns (Stratagene, La Jolla, CA) (for the libraries L02, L05, L10 and L11) or Dynabeads (Dynal, Oslo, Norway). The cDNA libraries were constructed using the UNI-ZAP^® ^XR Vector System (Stratagene, La Jolla, CA) following the manufacturer's instructions, excepting for the libraries L08 and L11, where the kit "Creator Smart cDNA library construction" (Clontech, Mountain View, CA) was used. In the latter case, it was performed a PCR in order to amplify the cDNA, before the cDNA cloning step.

### Clone isolation

*In vivo *excision of pBluescript^® ^plasmids from Uni-ZAP^® ^XR vector was performed in SOLR *Escherichia coli *host cells (Stratagene) following the manufacturer's instructions. Cells were plated on Q-Tray plates containing LB (Luria-Bertani) agar medium containing 100 μg/ml ampicillin and then, they were grown at 37°C overnight. Colonies were picked and then were distributed into known positions into 384-well plates, previously filled with 60 μl freezing medium per well, using a QPix robot (Genetix, New Milton, UK). One liter of freezing medium included: 900 ml of LB glycerol (10 g/l NaCl, 10 g tryptone, 5 g/l yeast extract and 44 ml/l glycerol), 100 ml of a solution composed by 90 ml of a mixture of salts (6.27 g K_2_HPO_4_, 1.80 g KH_2_PO_4_, 0.5 g trisodium citrate and 0.90 g ammonium sulphate, per each 90 ml) and 10 ml MgSO_4 _(0.1 g per each 10 ml), and 100 mg ampicillin. Well plates were incubated at 37°C overnight and then frozen at -80°C until used.

### DNA sequencing

Template DNA was extracted using a modified alkaline lysis protocol. Sequencing reactions were performed following standard Big Dye (Applied Biosystems, Foster City, CA) protocols for a 0.25X reaction. Cycle sequencing was performed over 35 cycles (96°C for 10 s; 50°C for 5 s; 60°C for 4 min) in an Applied Biosystems GenAmp 9700 thermocycler. Multiscreen 96-well plates (Millipore, Billerica, MA) were used for dye-terminator removal. The 5' end of each clone was sequenced using an ABI 3,100 capillary sequencer (Applied Biosystems).

### Sequence processing

The data were managed and stored using software specifically developed for the project. EST sequencing was performed and only the sequences containing more than 150 bases, with the program Phred [21] quality values greater than 20, were selected. Then, the EST sequences were cleaned using three programs included in the EMBOSS package [64]: Vectorstrip (for removing vector contamination), Trimseq (for removing the ambiguous ends of the sequences) and Trimest (for removing poly-A tails). Finally, the EST sequences were assembled into contigs using CAP3 [65]. Singlets and multisequence contigs resulting from this curation and assembly process were annotated on MySQL tables to build the TrichoEST database. A contig viewer written in PHP was used to browse the contigs. Several scripts in PHP and Python were used to parse the CAP3 standard output into MySQL tables. This method provided us with a clean table to keep exclusively unique sequences.

All unique sequences were queried against the NCBI non-redundant (nr) database (by January 2,006) and different datasets obtained for each species (Table 6) using the BLASTX algorithm [36] with default parameters. We also used the tBLASTX algorithm [36] with default parameters to compare our sequences with other collections of ESTs from *Trichoderma*. All unique sequences were submitted to InterProScan analysis [29] in order to search for protein motifs. A Java application was prepared to undertake the complex BLAST and InterPro analysis. This application was based in the BioJava library that was compatible with the API functions of a grid supercomputing facility available at our labs (based on the InnerGrid package, by GridSystems). Redundancy of the collections of ESTs was calculated as [1- (Number of unique sequences/Number of sequenced ESTs)] × 100. For this purpose, we only considered those sequenced ESTs that passed the quality criteria. Prediction of signal peptide cleavage sites was carried out by both the Hidden Markov Model and neural network modules of SignalP 3.0 [25].

### Assignment of GO terms

Annotations were based on the Gene Ontology (GO) terms and hierarchical structure [22]. The unigene set of EST contigs and singlets were annotated using the program Blast2GO [23,66] using the E-value < 10^-5 ^level. Blast2GO assigns the GO terms based on the BLAST definitions. The GO term annotations were merged and loaded into the AmiGO browser and database [67].

### Accession numbers

The nucleotide sequences of the generated ESTs were deposited in the EMBL database and have been assigned accession numbers from [EMBL:AJ893574] to [EMBL:AJ904494] inclusive. They are available as supplementary data in the additional files 1 and 3.

## Authors' contributions

JAV performed data analysis and drafted the manuscript. FJG constructed the TrichoEST database, designed the bioinformatics analysis and revised the manuscript. MBS and JR performed the construction of the cDNA gene libraries. JDJ designed growth conditions and revised the manuscript. JH performed the InterPro analysis and worked as grid sysadmin and developer. RH carried out the design of clone isolation and the automatic colony picking. EM, MR and AL were responsible for the design and implementation of the TrichoEST project and together with SG for the coordination and supervision of research in their respective laboratories. All authors read and approved the final manuscript.

## Supplementary Material

Additional file 1 Table of *T. harzianum *CECT 2413 unique sequences. It includes their name, accession number, BLAST annotation of the top hit, E-value and protein domains found in InterProScan analysis. The unique sequences that have putative signal peptides are highlighted in yellow. Click here for file

Additional file 2 Table of multisequence contigs and the ESTs that cluster in each of them. Click here for file

Additional file 3 Table of *T. harzianum *CECT 2413 unique sequences that are (i) common to fungi, plants and animals, (ii) fungal unique, (iii) common with fungi and animals but not present in plants and (iv) common with plants and fungi but not present in animals. It includes their name, accession number, BLAST annotation of the top hit, E-value and protein domains found in InterProScan analysis. Click here for file

## Figures and Tables

**Table 1:** cDNA library sources for sequences described in this study.

**Library ID**	**cDNA library name**	**Growth conditions**
L02	Walls presence	Cell walls from *P. syringae *and *B. cinerea*
L03	Mix 1	Glucose starvation, chitin, concentration of metals increased 100 times, cell walls from *P. digitatum *and *B. cinerea*.
L05	Glucose presence	Glucose
L06	Glucose starvation solid medium)	Glucose starvation in solid medium
L08	Colloidal chitin solid medium	Colloidal chitin in solid medium
L10	Mix 2	Chitin and nitrogen starvation
L11	*Rhizoctonia solani *confrontation	Confrontation *Trichoderma-R. solani *in solid medium
L15	Plant polymers	PGA, CMC, pectin, xylan and glucose

**Table 2:** Data of clustering and redundancy within the cDNA libraries.

**Library ID**	**Sequenced ESTs**	Quality ESTs *	**Singlets**	**Contigs**	**Unique sequences**
L02	3295	3056 (92.7%)	1064	656	1720
L03	1508	1330 (88.2%)	493	421	914
L05	192	185 (96.4%)	70	95	165
L06	739	486 (65.8%)	134	185	319
L08	192	187 (97.4%)	38	80	118
L10	1920	1434 (74.7%)	565	387	952
L11	480	290 (60.4%)	115	68	183
L15	384	315 (82.0%)	110	96	206
Combined	8710	7283 (83.6%)	2589	889	3478

* Phred [21] quality values greater than 20.

**Table 3:** Gene ontology (GO) functional assignments for the libraries L02, L03, L06 and L10.

**GO Term**	**GO ID**	**L02**	**L03**	**L06**	**L10**
**Biological Process**	GO:0008150	809 (100%)	389 (100%)	211 (100%)	472 (100%)
Cellular process	GO:0009987	738 (91.2%)	352 (90.5%)	196 (92.9%)	434 (91.9%)
Regulation of biological process	GO:0050789	57 (7.0%)	23 (5.9%)	12 (5.7%)	42 (8.9%)
Response to stimulus	GO:0050896	39 (4.8%)	29 (7.5%)	7 (3.3%)	31 (6.6%)
Physiological process	GO:0007582	778 (96.2%)	370 (95.1%)	211 (100%)	452 (95.8%)
Regulation of physiological process	GO:0050791	52 (6.4%)	20 (5.1%)	10 (4.7%)	36 (7.6%)
Metabolism	GO:0008152	647 (80.0%)	303 (77.9%)	196 (92.9%)	364 (77.1%)
Biosynthesis	GO:0009058	275 (34.0%)	139 (35.7%)	119 (56.4%)	153 (32.4%)
Catabolism	GO:0009056	73 (9.0%)	33 (8.5%)	6 (2.8%)	29 (6.1%)
Cellular metabolism	GO:0044237	580 (71.7%)	267 (68.6%)	181 (85.8%)	324 (68.5%)
Macromolecule metabolism	GO:0043170	392 (48.4%)	187 (48.1%)	136 (64.4%)	228 (48.3%)
Nitrogen compound metabolism	GO:0006807	90 (11.1%)	32 (8.2%)	17 (8.1%)	40 (8.5%)
Primary metabolism	GO:0044238	533 (65.9%)	249 (64.0%)	170 (80.6%)	301 (63.6%)
Regulation of metabolism	GO:0019222	42 (5.2%)	15 (3.9%)	7 (3.3%)	28 (5.9%)
Secondary metabolism	GO:0019748	11 (2.8%)	3 (0.8%)	0	2 (0.4%)

**Molecular Function**	GO:0003674	837 (100%)	389 (100%)	228 (100%)	483 (100%)
Binding activity	GO:0005488	400 (47.8%)	165 (42.4%)	107 (46.9%)	223 (46.2%)
Cofactor binding	GO:0048037	33 (3.9%)	15 (3.9%)	5 (2.2%)	16 (3.3%)
Ion binding	GO:0043167	78 (9.3%)	34 (8.7%)	12 (5.3%)	46 (9.5%)
Nucleic acid binding	GO:0003676	150 (17.9%)	65 (16.7%)	52 (22.8%)	82 (17.0%)
Nucleotide binding	GO:0000166	133 (15.9%)	57 (14.7%)	32 (14.0%)	61 (12.6%)
Protein binding	GO:0005515	54 (6.5%)	16 (4.1%)	18 (7.9%)	26 (5.4%)
Catalytic activity	GO:0003824	491 (58.7%)	232 (59.6%)	106 (46.5%)	284 (58.8%)
Hydrolase activity	GO:0016787	162 (19.4%)	81 (20.8%)	33 (14.5%)	119 (24.6%)
Lyase activity	GO:0016829	50 (6.0%)	23 (5.9%)	4 (1.8%)	15 (3.1%)
Ligase activity	GO:0016874	38 (4.5%)	18 (4.6%)	9 (3.9%)	17 (3.5%)
Oxidorreductase activity	GO:0016491	158 (18.9%)	71 (18.3%)	30 (13.2%)	78 (16.1%)
Transferase activity	GO:0016740	124 (14.8%)	50 (12.9%)	31 (13.6%)	56 (11.6%)
Transporter activity	GO:0005215	121 (14.5%)	57 (14.7%)	18 (7.9%)	80 (16.6%)
Carrier activity	GO:0005386	70 (8.4%)	32 (8.2%)	13 (5.7%)	49 (10.1%)
Ion transporter activity	GO:0015075	50 (6.0%)	22 (5.7%)	12 (5.3%)	36 (7.5%)
Signal transducer activity	GO:0004871	16 (1.9%)	7 (1.8%)	5 (2.2%)	13 (2.7%)
Structural molecule activity	GO:0005198	80 (9.6%)	47 (12.1%)	63 (27.6%)	41 (8.5%)
Enzyme regulator activity	GO:0030234	18 (2.2%)	13 (3,3%)	5 (2.2%)	12 (2.5%)
Transcription regulator activity	GO:0030528	17 (2.0%)	8 (2.1%)	5 (2.2%)	12 (2.5%)
Translation regulator activity	GO:0045182	52 (6.2%)	22 (5.7%)	21 (9.2%)	42 (8.7%)

**Cellular component**	GO:0005575	563 (100%)	280 (100%)	156 (100%)	316 (100%)
Extracellular region	GO:0005576	26 (4.6%)	18 (6.4%)	1 (0.6%)	21 (6.6%)
Cell	GO:0005623	539 (95.7%)	266 (95.0%)	151 (96.8%)	300 (94.9%)
Intracellular	GO:0005622	441 (78.3%)	211 (75.4%)	145 (92.9%)	235 (74.4%)
Membrane	GO:0016020	180 (32.0%)	97 (34.6%)	24 (15.4%)	112 (35.4%)

**Table 4:** The most abundantly represented genes.

**Contig ID**	**EST count**	**Annotation**	**E value**	**Libraries**
T34C463	95	Hydrophobin (*H. jecorina*)	8.00E-18	L02, L03, L05, L08, L10
T34C176	65	Unknown	N/A	L02, L03, L05, L10, L11
T34C29	56	Hypothetical protein (*Gibberella fujikuroi*) (contains InterPro Galactose binding domain)	2.00E-41	L02, L03, L05, L10, L11
T34C54	49	Hypothetical protein FG02077.1 (*G. zeae*) (contains InterPro CFEM domain, found in some proteins with a proposed role in fungal pathogenesis)	3.00E-19	L02, L03, L05, L06, L10, L15
T34C102	47	Hypothetical protein FG10224.1 (*G. zeae*) (contains phospholipase A2 active site)	7.00E-10	L02, L03, L10, L11
T34C494	46	NMT1_ASPPA NMT1 protein homolog (*G. zeae*)	E-123	L02, L03, L05, L10, L11
T34C278	45	Hypothetical protein FG05928.1 (*G. zeae*) (contains InterPro Zinc finger domain, found for example in transcription factors)	1.00E-09	L02, L03, L10, L15
T34C819	43	Hypothetical protein MG06538.4 (*Magnaporthe grisea*) (contains InterPro Bys1 domain, typical of the *Blastomyces *yeast-phase-specific proteins)	5.00E-50	L02, L03, L10, L11, L15
T34C760	42	Translation elongation factor 1α (*H. jecorina*)	E-109	L02, L03, L05, L06, L08, L10
T34C834	38	Hypothetical protein FG05928.1 (*G. zeae*) (contains InterPro Zinc finger domain)	2,00E-09	L02, L03, L10, L11
T34C311	32	Hypothetical protein FG10224.1 (*G. zeae*) (contains phospholipase A2 active site)	7.00E-10	L02, L03, L05, L10, L11
T34C43	32	Translation elongation factor 1α (*H. jecorina*)	E-120	L02, L03, L06, L10
T34C508	31	Hypothetical protein FG01267.1 (*G. zeae*)	1.00E-30	L02, L03, L10
T34C848	31	Translation elongation factor 1α (*H. jecorina*)	E-102	L02, L03, L05, L10, L15
T34C748	29	Histone H4 (*A. nidulans*)	4.00E-38	L02, L03, L06, L10
T34C195	28	Polyubiquitin (*A. fumigatus*)	E-166	L02, L03, L06, L10, L15
T34C470	28	ADP/ATP carrier protein (*N. crassa*)	E-101	L02, L03, L05, L06, L10, L15
T34C811	28	THI4_FUSOX Thiazole biosynthetic enzyme, mitochondrial precursor (Stress-inducible protein sti35) (*G. zeae*)	1.00E-84	L02, L03, L05, L08, L10, L11
T34C829	24	NMT1_ASPPA NMT1 protein homolog (*G. zeae*)	1.00E-87	L02, L03, L10, L11
T34C273	24	Histone H3 (*H. jecorina*)	8.00E-69	L02, L03, L06, L10, L15
T34C180	23	Unknown	N/A	L02, L03, L10, L11, L15
T34C216	23	ATP9_NEUCR ATP synthase protein 9, mitochondrial precursor (Lipid-binding protein) (*G. zeae*)	3.00E-44	L02, L05, L06, L10
T34C168	22	Glyceraldehyde 3-phosphate dehydrogenase (*Trichoderma koningii*)	E-102	L2, L3, L5, L10
T34C759	22	Putative stress response RCI peptide (*A. fumigatus*)	3.00E-21	L02, L03, L10, L15
T34C292	20	60 S acidic ribosomal protein P2 (*Alternaria alternata*)	2.00E-18	L02, L03, L05, L06, L10
T34C418	20	Cyclophilin, cytosolic form (*Tolypocladium inflatum*)	2.00E-73	L02, L03, L05, L06, L08, L10
T34C846	20	hypothetical protein FG10150.1 (*G. zeae*)	4.00E-35	L02, L03, L05, L10, L15

**Table 5:** Comparison of the *T. harzianum *unique sequences against complete sequenced genomes from different animals, plants and fungi.

**Organism**	**e < 10^-5^**	**e < 10^-25^**	**e < 10^-50^**	**e < 10^-75^**	**e < 10^-100^**
*A. nidulans*	2055 (59.1%)	1302 (37.4%)	689 (19.8%)	283 (8.1%)	88 (2.5%)
*A. gossypii*	1327 (38.2%)	706 (20.3%)	325 (9.3%)	115 (3.3%)	38 (1.1%)
*C. albicans*	1439 (41.4%)	785 (22.6%)	354 (10.2%)	130 (3.7%)	39 (1.1%)
*C. neoformans*	1248 (35.9%)	650 (18.7%)	279 (8.0%)	95 (2.7%)	33 (0.9%)
*F. graminearum*	2494 (71.7%)	1787 (51.4%)	1103 (31.7%)	552 (15.9%)	199 (5.7%)
*M. grisea*	2205 (63.4%)	1450 (41.7%)	805 (23.1%)	359 (10.3%)	106 (3.0%)
*N. crassa*	2311 (66.4%)	1515 (43.6%)	859 (24.7%)	398 (11.4%)	140 (4.0%)
*S. cerevisiae*	1355 (39.0%)	721 (20.7%)	347 (10.0%)	112 (3.2%)	40 (1.2%)
*S. pombe*	1363 (39.2%)	748 (21.5%)	342 (9.8%)	123 (3.5%)	39 (1.1%)
*T. reesei*	2616 (75.2%)	2160 (62.1%)	1550 (44.6%)	962 (27.7%)	403 (11.6%)
*U. maydis*	1492 (42.0%)	783 (22.5%)	334 (9.6%)	117 (3.4%)	36 (1.0%)
Fungi	2790 (80.2%)	2281 (65.6%)	1628 (46.8%)	1006 (28.9%)	436 (12.4%)

*A. thaliana*	1129 (32.5%)	493 (14.2%)	192 (5.5%)	66 (1.9%)	17 (0.5%)
*O. sativa*	1018 (29.3%)	449 (12.9%)	177 (5.1%)	64 (1.8%)	14 (0.4%)
Plants	1158 (33.3%)	506 (14.5%)	196 (5.6%)	67 (1.9%)	17 (0.5%)

*C. elegans*	872 (25.1%)	385 (11.1%)	153 (4.4%)	53 (1.5%)	15 (0.4%)
*D. melanogaster*	897 (25.8%)	408 (11.7%)	161 (4.6%)	62 (1.8%)	19 (0.5%)
Animals	1087 (31.3%)	491 (14.1%)	188 (5.4%)	70 (2.0%)	20 (0.6%)

All organisms	2793 (80.3%)	2287 (65.8%)	1632 (46.9%)	1007 (29.0%)	430 (12.4%)

**Table 6:** Genomic datasets used in this study.

**Species**	**Taxa**	**Data origin**
*Aspergillus nidulans*	Ascomycete	Broad Institute. Data as of 07/03/2003 [69].
*Ashbya gossypii*	Ascomycete	*Ashbya *genome database. Data as of release 2.1 [70].
*Arabidopsis thaliana*	Plant	The TIGR *Arabidopsis thaliana *database. Data as release 5.0 [71].
*Caenorhabditis elegans*	Animal	The worm database. Data as of v.150 [72].
*Candida albicans*	Ascomycete	The *Candida *genome database. Data as of 16/7/2005 [73].
*Cryptococcus neoformans*	Basidiomycete	Stanford University. Data as of v.040623 [74].
*Drosophila melanogaster*	Animal	Flybase. Data as of release 4.2.1 [75].
*Fusarium graminearum*	Ascomycete	Broad Institute. Data as of 11/03/2003 [76].
*Magnaporthe grisea*	Ascomycete	Broad Institute. Data as of 31/10/2003 [77].
*Neurospora crassa*	Ascomycete	Broad Institute. Data as of release 7 [78].
*Oryza sativa*	Plant	The TIGR rice genome annotation. Data as of release 3.0 [79].
*Saccharomyces cerevisiae*	Ascomycete	*Saccharomyces *genome database. Data as of 27/8/2004 [80].
*Schizosaccharomyces pombe*	Ascomycete	The Sanger Institute. Data as of 18/8/2005 [81].
*Trichoderma reesei*	Ascomycete	The Joint Genome Institute. Data as v1.2 predicted proteins [82].
*Ustilago maydis*	Basidiomycete	Broad Institute. Data as of 01/04/2004 [83].
